# Effect of Particle Size and Support Type on Pd Catalysts for 1,3-Butadiene Hydrogenation

**DOI:** 10.1007/s11244-018-0887-4

**Published:** 2018-01-19

**Authors:** Donato Decarolis, Ines Lezcano-Gonzalez, Diego Gianolio, Andrew M. Beale

**Affiliations:** 1 Chemistry Department, University College London, 20 Gordon Street, London, WC1H 0AJ UK; 2 Rutherford Appleton Laboratory, Research Complex at Harwell, Harwell Science and Innovation Campus, Harwell Didcot, Oxon, OX11 0FA UK; 3 Diamond Light Source Ltd, Harwell Science and Innovation Campus, Chilton, Didcot, OX11 0DE UK

**Keywords:** Palladium, Butadiene, Particle size, Hydrogenation

## Abstract

Pd nanoparticles supported on SiO_2_, Si_3_N_4_ and Al_2_O_3_ were studied to examine the effect of particle size and support type on the hydrogenation of 1,3-butadiene. Pd nanoparticles were produced using a reverse micelle method resulting in particles with a remarkably small particle size distribution (σ < < 1 nm). The support type and particle size were observed to affect both catalytic activity and product selectivity. All catalysts showed a decrease of their activity with time on stream, paired with an increase in selectivity to butenes (1-butene and *cis*/*trans*-2-butene) from a product stream initially dominated by *n*-butane. In situ XAFS demonstrated a correlation between the formation of palladium hydride and *n*-butane production in the early stages (~ 1 h) of reaction. The extent of palladium hydride formation, as well as its depletion with time on stream, was dependent on both particle size and support type. Metallic Pd was identified as the species selective towards the production of butenes.

**Electronic supplementary material:**

The online version of this article (10.1007/s11244-018-0887-4) contains supplementary material, which is available to authorized users.

## Introduction

Supported palladium nanoparticles (Pd NPs) have shown excellent ability to selectively hydrogenate unsaturated hydrocarbons, of importance in the petrochemical, pharmaceutical and fine chemical industries. They are widely employed in the purification of alkene streams to remove traces of alkynes and dienes, such as 1,3-butadiene—a common impurity in C_4_ cracking streams, which may negatively affect downstream polymer units. While this is nowadays a standard process, it is limited by the tendency of Pd to hydrogenate the desired olefins and in particular to isomerise those olefins containing more than three carbon atoms [1]. The reaction mechanism for the hydrogenation of unsaturated C–C bonds was proposed by Horiuti and Polanyi [2], involving hydrogen dissociation on the metal surface followed by alkene adsorption, hydrogen addition to the alkene, and desorption of the product. Previous studies have shown that Pd exhibits a high activity for molecular hydrogen splitting, as well as the ability to absorb hydrogen into its bulk [3].The hydrogen dissolved in the bulk forms a new phase, palladium hydride, an intermetallic species where hydrogen atoms occupy interstitial octahedral vacancies in the palladium lattice. Debate arises regarding the role of different species of hydrogen on palladium, surface and bulk, with bulk hydrogen atoms proposed to be key for the hydrogenation of olefins [4].

As with most nanoparticulate systems, the size of Pd NPs is a key factor in catalytic performance. For example, Tardy et al. showed a particle size dependence for the 1,3-butadiene hydrogenation; i.e. while particles with a diameter larger than 2.8 nm behave like bulk Pd, below this value a size-effect is observed, especially when the particle size is between 2.8 and 1.4 nm. This behaviour was explained by deactivation due to strong butadiene adsorption; in addition some change may occur due to a geometric effect, and structural properties which can be induced by an epitaxial strain of the nanoparticles [5]. In addition, Pd has shown structure sensitivity for hydrocarbon hydrogenation, with Pd (110) faces fivefold more active than Pd (111) for single crystal catalysts [6]. Pd nanoparticles usually exhibit facets of different crystallographic orientations so that variation in the relative abundance of the different facets with particle size has also an effect on the catalytic activity [5].

In a study from Silvestre-Albero et al. it was shown that Pd NPs were not always perfect cuboctahedra, presenting incomplete (111) and (100) terraces. STM results revealed that small (< 3.5 nm) metal clusters were highly defective, with a slight increase in particle size leading to an increase in the proportion of defects at the boundary of the incomplete facets. In contrast, particles > 3.5 nm show large and well defined (111) and (100) facets [6, 7]. Based on normalized TOF values, the authors concluded that the selective hydrogenation of 1,3-butadiene is particle size independent for Pd particles ≥ 4 nm. For Pd particles < 4 nm, the normalisation was not straightforward due to the highly defective nature of the small nanoparticles however, TOF values approached those of the Pd (110) single crystal when it was calculated considering a nanoparticle model based on a cuboctahedron. Accordingly, the relative greater abundance of surface defects may allow for an enhanced hydrogen penetration, giving rise to greater-than-expected activity [6, 7].

In a recent study, Dal Santo et al. showed that a combination of both particle size and morphology of Pd NPs (the exposed planes) affects the catalytic behaviour for 1,3-butadiene hydrogenation. In their study they prepared two sets of supported NPs on different oxides through chemical vapour deposition, with different pre-treatment of the support. The pre-treated supports gave rise to highly dispersed NPs as compared to the non-treated sample, showing an overall higher selectivity to butenes [8]. According to Lee et al. selectivity to butenes can be controlled through a modification of the surface properties of Pd such as decoration with TiO_x_ species [9]. Partially reduced TiO_x_ species migrate onto and decorate Pd surfaces, significantly suppressing H_2_ uptake and leading to a lower hydrogenation rate for butenes. Furthermore, selectivity can be also controlled by the suppression of π-allylic species, formed preferentially on a large ensemble surface of Pd. Goetz et al. [10], showed that two types of structure were formed depending on the Pd concentration on the Al_2_O_3_ support; i.e. flat (111) surfaces (Pd = 0.1%) and particles with less interaction with the support (10) (Pd = 0.3%). Two reaction mechanisms for isomerisation of 1-butene to 2-butenes species, and vice versa, were identified: a Horiuti–Polanyi mechanism involving the addition of hydrogen atoms to form a half-hydrogenated radical and an intramolecular hydrogen shift mechanism which occurs without addition of hydrogen, and a Horiuti–Polanyi mechanism occurring on corner and edge atoms, with the hydrogen shift thought to occur on face atoms. Accordingly, reduction of the Pd ensemble surfaces by decoration with TiO_x_ species leads to a decrease in the isomerisation rate of 1-butene, also facilitating its desorption [9].

The main aim of this work lies in understanding particle size and support effects for Pd NPs on the selective hydrogenation of 1,3-butadiene. Information regarding particle size and support effects is paramount to improve the catalytic activity of nanoparticle catalysts, though production of highly homogeneous particles is necessary to obtain meaningful insight on the role of the particle size on the catalytic activity. A problem arises however when examining catalysts prepared using different methods; the use of different precursors, catalyst supports or preparation conditions leads to catalysts which were intrinsically different besides a difference in particle size [11]. For example, methods of preparation such as impregnation may cause a large particle size distribution, as seen in a not atypical paper by Okumura et al. where a standard deviation of ~ 50% of the total gold nanoparticles were present [12]. This might cause problems in the identification of the real active part of the particle size due the possible interference of larger and smaller nanoparticle, or even atomically dispersed gold such as in the case of a study from Rogers et al. [13]. Thus, methods such as reverse micelle encapsulation, developed by Spatz et al. [14] and later employed to produce monodisperse nanoparticles [15, 16], are very useful for obtaining meaningful results regarding the role of particle size in catalysis. We recently used this method, which produces nanoparticles with a very narrow particle size distribution (σ < < 1 nm), to understand the restructuring process of Au nanoparticles under reaction conditions [17]. In this work, the reverse micelle encapsulation method has been employed to prepare homogeneously-sized Pd NPs and these have been characterised by in situ XAFS spectroscopy in order to identify any modification caused by adsorption and interaction of the reaction gases (H_2_, 1,3-butadiene and the mixture of the two during the hydrogenation of 1,3-butadiene). Correlation between the catalytic data obtained for 1,3-butadiene hydrogenation and the characterisation results allows for obtaining a deeper understanding into the effect of both particle size and type of support on the selective hydrogenation of 1,3-butadiene.

## Materials and Methods

### Catalyst Synthesis

Palladium nanoparticles were synthesised using the reverse micelle method pioneered by Spatz et al. [14]. The polymers used were:

- P4708-S2VP (polystyrene (PS) = 16,000 MW, poly-2 vinylpyridine (P2VP) = 3500 MW), polydispersity = 1.05;
- P18226-S2VP (polystyrene (PS) = 30,000 MW, poly-2 vinylpyridine (P2VP) = 8500 MW), polydispersity = 1.06,
- PS5073-S2VP (polystyrene (PS) = 175,000 MW, poly-2 vinylpyridine (P2VP) = 70,000 MW, polydispersity = 1.08),

all from Polymer Source Inc.; the purity 100% in all cases.

The metal salt used was potassium tetrachloropalladate (Aldrich 99.99999% trace metal basis). The metal atom-to-pyridine ratio MS:Pyr (metal loading) was fixed at 0.3 MS:Pyr for all the samples while the reducing agent used was p-Toluene Sulfonyl hydrazide (p-Tosyl hydrazide) (97% Aldrich). In a typical preparation a 50 ml, 0.5 wt% (c = 5 mg/ml) solution was prepared and mixed for 3 days in order to allow the polymer to dissolve and homogenise. Subsequently, the metal precursor was added to the solution, the amount dependent on the polymer used (0.053 g for P4708-S2VP, 0.042 g for P18226-S2VP and 0.069 g for PS5073-S2VP, respectively) and left to incorporate inside the micelles. After a further 3 days stirring, an aliquot of P-Tosyl hydrazide (fourfold the concentration of metal) was dissolved in 50 ml of toluene and added to the solution and, ~ 3 min later, 0.5 ml of HCl (ACS reagent 37%, Fluka) was added to the solution. After further ~ 10 min. the support, in powder form, was added to the solution.

Al_2_O_3_ (Alfa Aesar, 99.9%; 100 m^2^/g), SiO_2_ (Alfa Aesar; 85–113 m^2^/g) and Si_3_N_4_ (Alfa Aesar, 98.5%; 103–123 m^2^/g) were used as supports. These were added to the solution in order to obtain a 1% concentration of metal and left to stir-dry in air overnight (i.e. until all toluene evaporated). The samples were then calcined at 500 °C, in static air for 2.5 h in an alumina crucible and subsequently characterised.

### Catalyst Characterisation

The various samples have been characterised by transmission electron microscopy (TEM), using a JEOL: JEM-2100 operating at 200 keV with a LaB_6_ filament, in order to define the particle size and the particle size distribution. A small amount of sample was dissolved in ethanol and subjected to sonication to allow dispersion. Subsequently a few drops of the resulting solution were deposited onto a holey carbon film supported on a 300 mesh copper TEM grid.

### Catalyst Testing

The prepared samples were tested in the hydrogenation of 1,3-butadiene. The catalysts (~ 275 mg for Al_2_O_3_, and Si_3_N_4_ supported samples, ~ 165 mg for SiO_2_ supported samples), with a sieve fraction of 250–425 µm, were loaded inside a reactor tube (7 mm diameter), and then placed inside a tubular furnace and connected with gas lines through Swagelok fittings. The samples were initially treated in H_2_ in order to reduce PdO to Pd^0^ (30 min under H_2_ atmosphere at 323 K). After treatment, the catalysts were brought to room temperature (~ 298 K) and butadiene hydrogenation was performed using a space velocity of 22,500 h^−1^, using a reaction mixture composed of hydrogen (4% in Helium, BOC Ltd.) and 1,3-butadiene (1% in Helium, BOC Ltd.) in a percentage ratio of 95.2/4.8, using additional Helium for balance; the flow, in ml/min, was 150 H_2_, 30 butadiene, 225 He respectively. The activity and selectivity was monitored using a mass spectrometer (MS) and a gas chromatograph (GC). The gas chromatograph was calibrated using a calibration mixture comprising (0.05% *cis*-2-butene, 0.15% *trans*-2-butene, 0.1% *N*-butane, 0.3% 1-butene all balanced in He, BOC). For all catalysts tested a first GC measurement was performed 10–15 s after the online MS was able to detect the first reaction products.

### In Situ XAFS

The measurements were performed in B18 beamline at Diamond Light Source at the palladium *K* edge (24.35 keV) in transmission mode, using 75.7 s per scan. The catalysts, with a sieve fraction of 125–250 μm, were loaded into a capillary and mounted on a catalyst test rig with the capillary connected to gas lines on one side and a residual gas analyser (MS) on the other. While under He flow, the catalysts were brought to 160 °C and subsequently different gases, 10% H_2_ in He, 1% 1,3-butadiene in He or a mixture of the two (2:98 in relative %) were passed over the catalysts. XAFS spectra were recorded after keeping the samples under steady state for 20 min at each gas composition. In order to verify the reversibility of the system, hydrogen (10% in He) was passed over the catalyst after completion of the reaction. Data processing and analysis were done using Athena and Artemis software from the Demeter IFEFFIT package [18, 19]. The FEFF6 code was used to construct theoretical EXAFS signals that included single-scattering contributions from atomic shells through the nearest neighbours in the face-centered cubic (FCC) structure of Pd, up to the fourth coordination shell. The *k*-range used for the fitting 3–12 Å^−1^ and the *r*-range from 1.15 to 3 Å. The path degeneracy was allowed to vary in the fit in order to account for the size effects that cause surface atoms to be less coordinated than those in the particle interior. The amplitude reduction factor (S^2^_0_) was fixed at 0.860, as obtained from the fitting of the bulk Pd foil. Due to the high correlation between Debye–Waller factor (DWF) and first shell coordination number (1CN) (> 0.95) the fitting leads to a high error; the results obtained do not match with the variation that can be seen from the Fourier transform of the EXAFS data taken at different gas composition. Therefore, the DWF has been fixed to the results obtained by fitting the sample under 1,3-butadiene atmosphere.

## Results

### Catalyst Characterisation

**Fig. 1:**
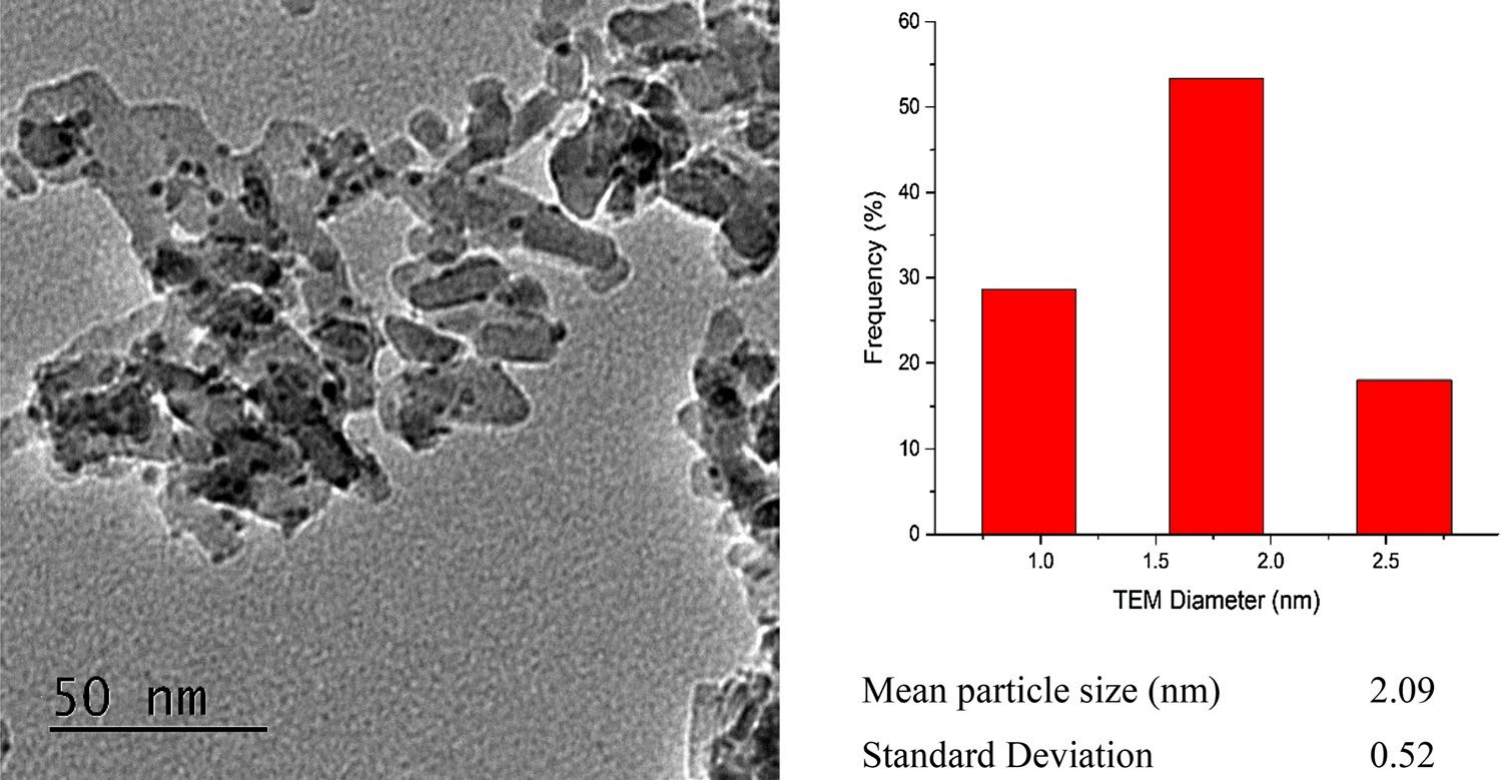
Left: representative TEM micrograph of Pd/Al_2_O_3_ 30-8.5 sample after synthesis. Right: summative particle size distribution (total 150 particles counted)

The TEM results are shown in Fig. 1, S1 and S2. The average particle size was obtained by analysing 150 nanoparticles (on average 5–10 images) taken from different parts of the catalyst in order to obtain a representative overview of the particle size distribution. As can be seen from Figs. S1 and S2, for SiO_2_ and Si_3_N_4_ overlap of the support particles does not allow a clear identification of the Pd NPs even considering the higher contrast they produce (due to the higher Z of Pd compared to the other elements). In general, it appears the nanoparticles present themselves as spherical. It appears that the nanoparticles show a low dispersion in particle size, with the standard deviation being < < 1 nm; moreover, nanoparticles which were produced using the same polymer appear to present a similar particle size, evidence that the method can be applied when using different supports.

In Table 1 are shown the TEM results obtained for all samples which provides an illustration of the ‘number averaged’ particle size, together with the particle size diameter obtained from EXAFS analysis which provides a particle size based on a bulk average [20]. In general, there is a good correlation between the two measurements with mostly < 1 nm difference. However, the particle size observed by EXAFS was always greater than that reported for TEM. This difference between the TEM and EXAFS results could be explained by the presence of larger Pd nanoparticles, not seen in the TEM area examined, thus increasing the Pd–Pd contribution in the EXAFS. We propose that since we did not observe any particles larger than the mean value, those which were present were few and large and therefore not likely to influence catalytic activity particularly.

**Table 1:** List of samples and their mean particle size obtained from TEM and EXAFS, respectively

Sample	Polymer used	Particle size TEM (nm)	Particle size EXAFS (nm)
Pd on SiO_2_ (16-3.5)	P4708-S2VP	1.1 ± 0.3	2.4 ± 0.24
Pd on SiO_2_ (30-8.5)	P18226-S2VP	1.9 ± 0.3	2.7 ± 0.27
Pd on SiO_2_ (175-70)	PS5073-S2VP	2.6 ± 0.5	3.2 ± 0.32
Pd on Al_2_O_3_ (30-8.5)	PS5073-S2VP	2.1 ± 0.5	1.1 ± 0.1^a^
Pd on Si_3_N_4_ (30-8.5)	PS5073-S2VP	2.1 ± 0.5	3 ± 0.3

^a^The value obtained from the fit could be influenced by the presence of PdH which lowers the amplitude of the EXAFS and therefore of the 1CN

### Catalytic Performance at 298 K

#### Effect of the Support

**Fig. 2:**
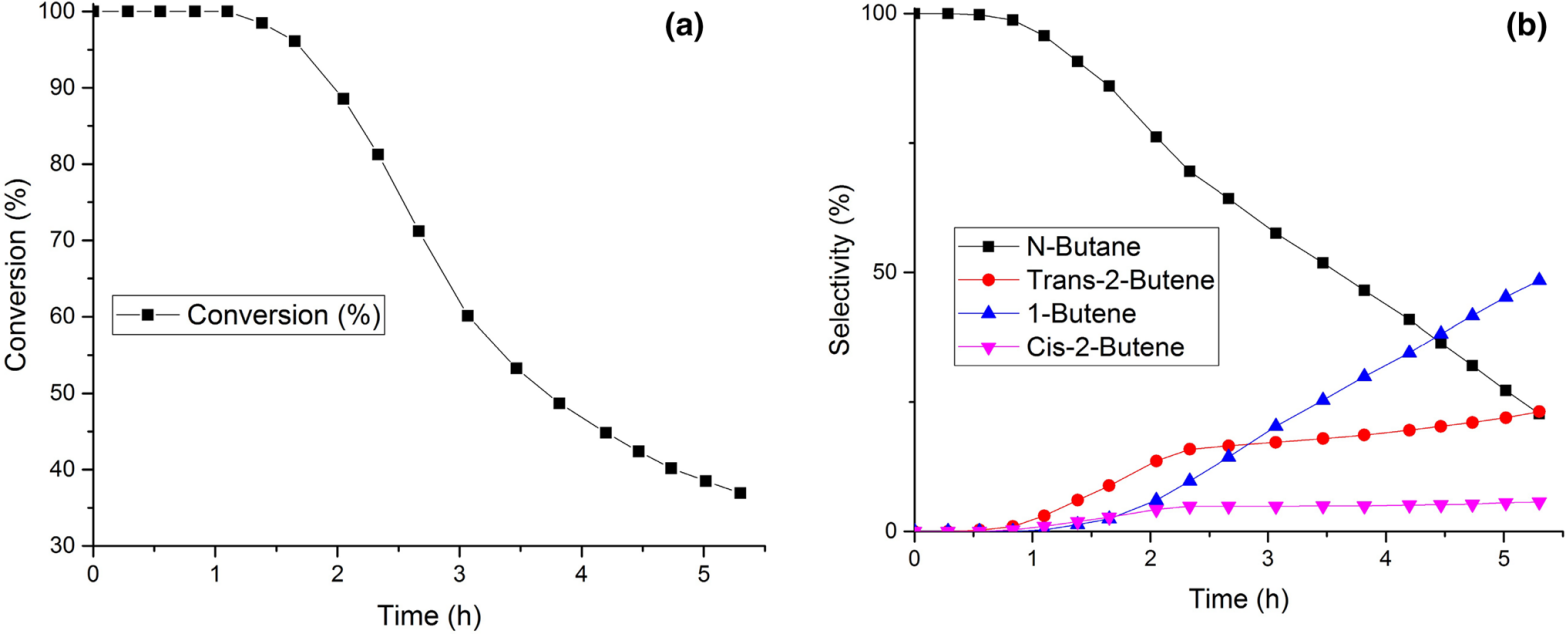
Evolution of conversion (**a**) and selectivity (**b**) for 1,3-butadiene hydrogenation over Pd/Al_2_O_3_ 30-8.5 as a function of time on stream

The catalytic performance of the samples was investigated in the hydrogenation of 1,3-butadiene in order to get further insight into the role of the support and the effect of particle size of Pd NPs. As seen in Fig. 2, the Pd/Al_2_O_3_ 30-8.5 sample converted completely 1,3-butadiene to butane for the first hour of reaction. With time on stream (TOS) a decrease in conversion was observed (Fig. 2a). As the activity decreased the product composition changed, as seen by a continuous drop in butane formation accompanied by an increase in butene selectivity. The cause for this is likely to be due to a depletion of volume-adsorbed hydrogen which was supposed to be the responsible for over-hydrogenation [21]. After 4 h of reaction the main product detected was 1-butene, with minor amounts of *trans*- and *cis*-2-butene also being produced. This product distribution was commonly seen in literature but there was no explanation regarding its cause [7, 22]. The production of 1-butene was attributed to a 1,2-hydrogenation whereas the 1,4-hydrogenation appeared to produce *cis*/*trans*-butenes. However the way these isomers are produced depends on the structure of the adsorption layer and the availability of hydrogen [23]. In order to compare the catalytic performance of the different samples studied, all catalytic reactions were allowed to continue until no further drop in conversion occurred. For Pd/Al_2_O_3_ 30-8.5 the reaction was stopped after 5.3 h due its high activity which did not allow the sample to completely deactivate.

**Fig. 3:**
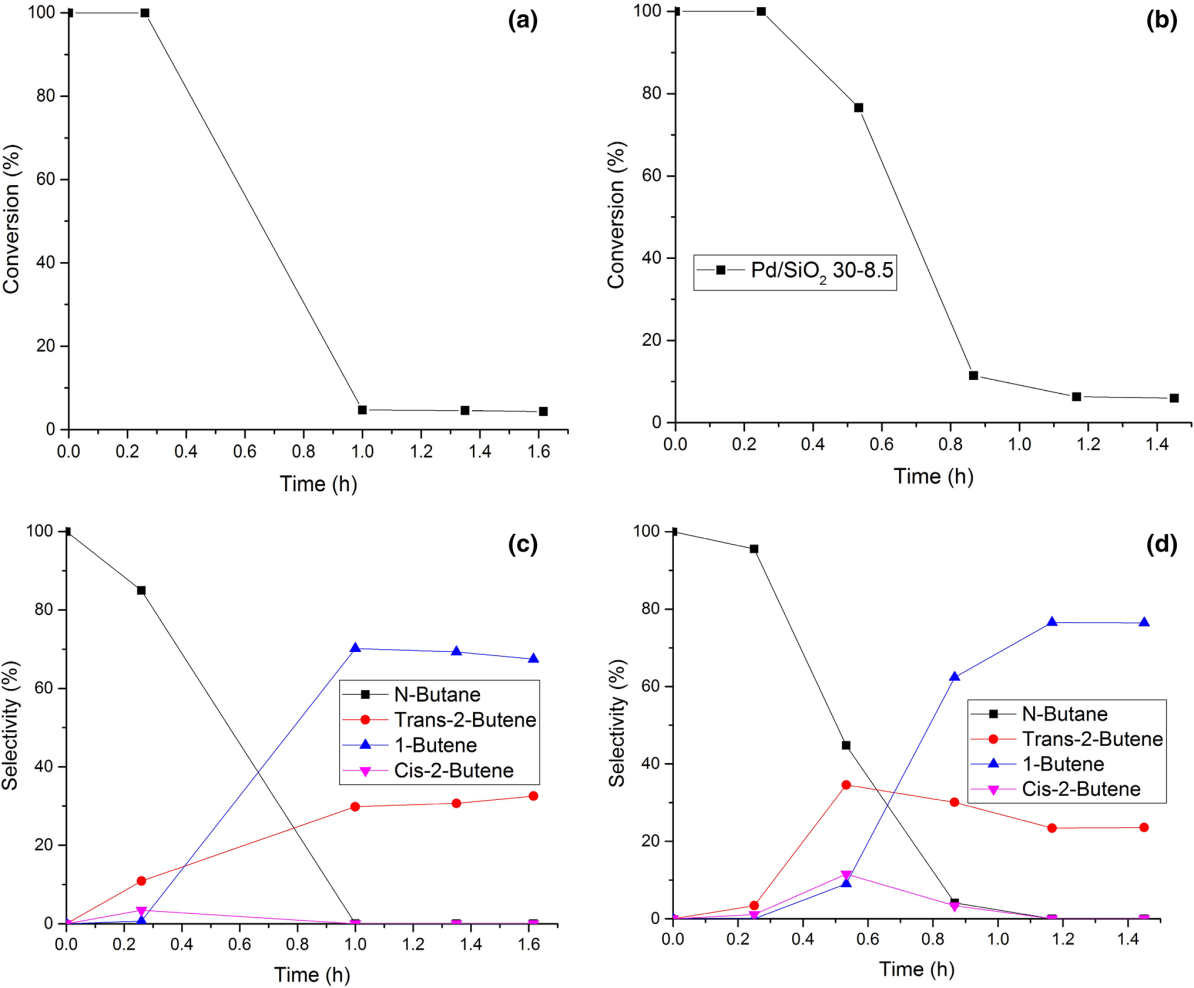
Evolution of conversion (**a, b**) and selectivity (**c, d**) for 1,3-butadiene hydrogenation over Pd/Si_3_N_4_ 30-8.5 (**a, c**) and Pd/SiO_2_ 30-8.5 (**b, d**) as a function of time on stream

**Fig. 4:**
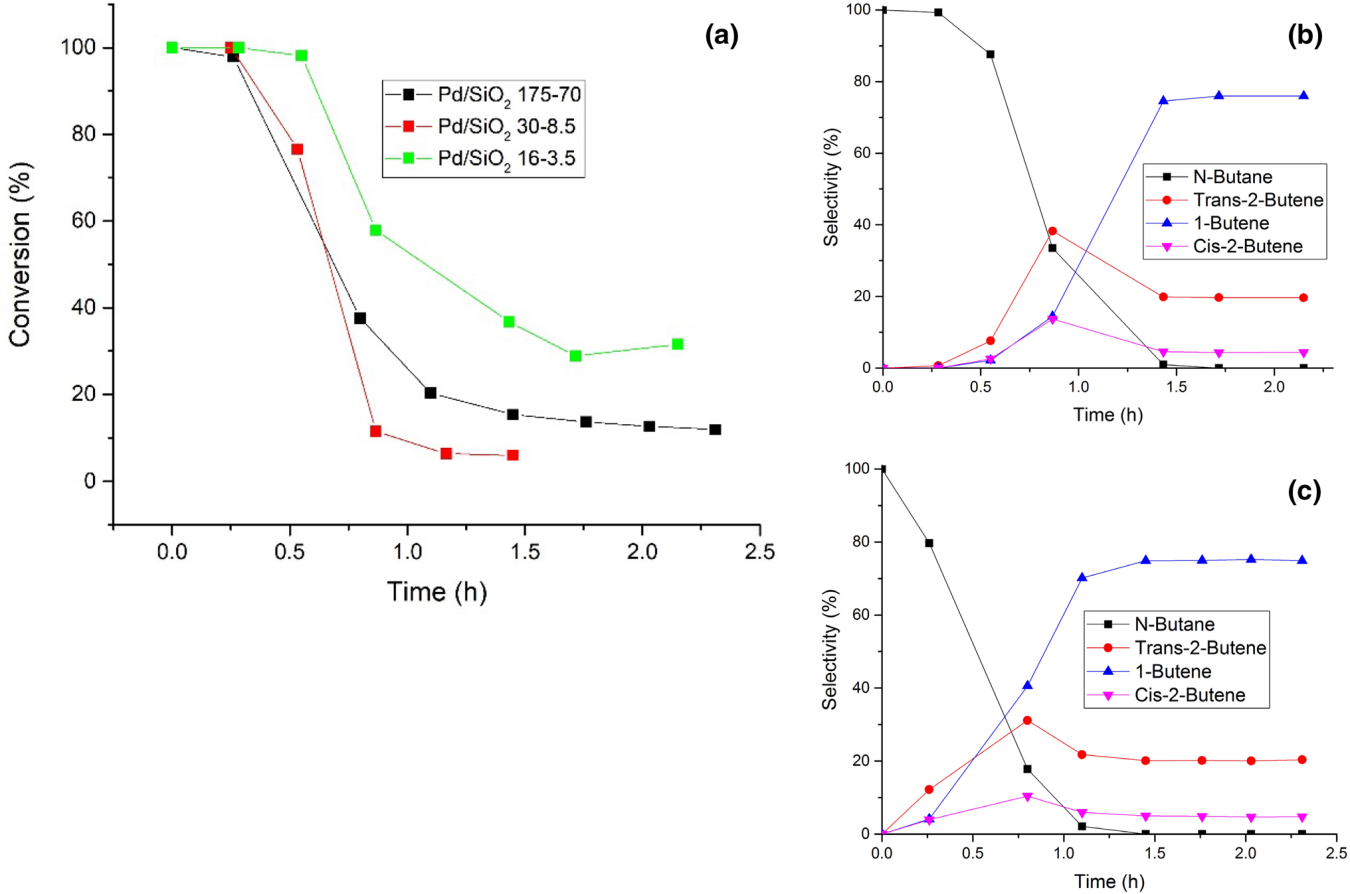
Evolution of conversion (**a**) and selectivity [**b** (Pd/SiO_2_ 16-3.5), **c** (Pd-SiO_2_ 175-70)] for 1,3-butadiene hydrogenation over Pd/SiO_2_ catalysts as a function of time on stream. The particle size for the Pd/SiO_2_ catalysts were 1.1, 1.9, 2.6 nm for Pd/SiO_2_ 16-3.5, Pd/SiO_2_ 30-8.5 and Pd/SiO_2_ 175-70, respectively

The Pd/Si_3_N_4_ 30-8.5 sample (Fig. 3) deactivated much more quickly than Pd/Al_2_O_3_ 30-8.5, reaching a consistently low level of conversion after 1 h; a consequence of this was an increase in selectivity to *1*-butene. In Fig. 4 is shown the catalytic performance of Pd/SiO_2_ 30-8.5. The deactivation of the catalyst and the selectivity profile for this material appears to proceed similarly to the one reported for the Si_3_N_4_ supported sample shown in Fig. 3. As can be seen from Table 2 the type of support appears to affect the activity considerably. After 1.4 h, Pd/Al_2_O_3_ 30-8.5 showed the highest conversion amongst the samples examined (98% total conversion) but with a low selectivity towards butenes (9.25%). The samples supported on SiO_2_ and Si_3_N_4_ performed worse in terms of conversion, (12.65 and 4.5% respectively) but the selectivity to butenes was higher than for the Al_2_O_3_ supported sample, with no formation of *n-*butane; moreover, no formation of *cis*-2-butene was observed. This could be explained either by the absence of facets where the adsorption of the 1,3-butadiene in a *cis*-configuration allows the 1,4-hydrogenation to happen, as well as a fast isomerisation process skewed towards the production of *trans*-2-butene. After 5.3 h of reaction, the Al_2_O_3_ supported sample conversion dropped to 37%, drastically improving its selectivity towards butenes (from 9.25 to 77.3%).

**Table 2:** Catalytic activity/selectivity comparison between samples with similar Pd particle size but different support, SiO_2_, Si_3_N_4_ and Al_2_O_3_ at 1.4 h time on stream

Sample	Conversion (%)	*n*-Butane selectivity (%)	*trans*-2-butene selectivity (%)	1-butene selectivity (%)	*cis*-2-butene selectivity (%)
Pd/Al_2_O_3_ 30-8.5	98.5	90.7	6.0	1.3	1.9
Pd/Si_3_N_4_ 30-8.5	4.3	0	32.6	67.4	0
Pd/SiO_2_ 30-8.5	6.0	0	23.6	76.4	0

#### Effect of Particle Size

**Fig. 5:**
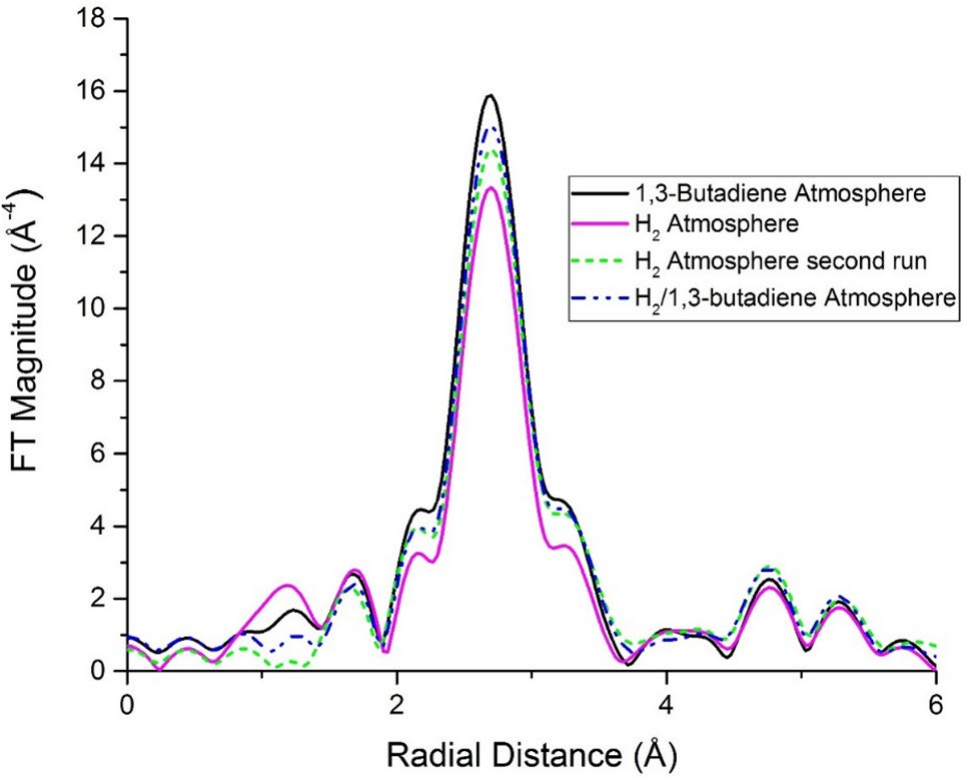
k^3^-weighted Fourier transform of Pd/SiO_2_ 30-8.5, under hydrogen and 1,3-butadiene and the mixture of the two (H_2_/1,3-butadiene ratio 49/1 during reaction). As the gas composition changes, a change in intensity of the first Pd–Pd scattering shell (~ 2.7 Å) is seen as a result of the ‘gas interaction’ with the Pd nanoparticles

SiO_2_ supported samples were used to investigate the particle size effect of Pd on 1,3-butadiene hydrogenation. The three samples appeared to show that a particle size effect was indeed present. In Fig. 5 are shown the catalytic profiles for the SiO_2_ supported samples, summarised in Table 3 for clarity. The Pd/SiO_2_ 30-8.5 sample presented the lowest activity amongst the SiO_2_ supported catalysts tested (12.65% conversion), with Pd/SiO_2_ 16-3.5 presenting the highest activity overall (31.57% conversion). These results show an unexpected reverse volcano plot (i.e. with respect to particle size). The selectivity for all the samples were similar; both Pd/SiO_2_ 16-3.5 and 175-70 (1.1 and 2.6 nm respectively) showed the production of *cis-*2-butene after 1.5 h, possibly due to isomerisation from *trans*-2-butene since the amount of 1-butene obtained from all the reaction was consistently ~ 75% of the total product.

**Table 3:** Catalytic activity/selectivity comparison between samples supported on SiO_2_ but with different particle size (1.1, 1.9 and 2.7 nm, respectively) after 1.5 h

Sample	Conversion (%)	*n*-Butane selectivity (%)	*trans*-2-butene selectivity (%)	1-butene selectivity (%)	*cis*-2-butene selectivity (%)
Pd/SiO_2_ 16-3.5	31.6	0	19.6	75.9	4.4
Pd/SiO_2_ 30-8.5	6.0	0	23.6	76.4	0
Pd/SiO_2_ 175-70	11.9	0	20.4	74.9	4.7

### Catalytic Performance at 353 K

#### Effect of the Support

The catalysts were tested following the same procedure at 353 K in order to replicate the conditions present during XAFS data acquisition. Pd/Al_2_O_3_ 30-8.5 was tested for 6 h but no decrease in conversion was observed in contrast to the data collected at room temperature. The only product was *n*-butane even after 6 h of time on stream. Pd/SiO_2_ 30-8.5 (S3a) on the other hand produced no *n*-butane throughout the reaction process. Compared to room temperature, the catalytic conversion increased, from ~ 6 to ~ 20% as shown in Table 4. Interestingly, the behaviour of Pd/Si_3_N_4_ 30-8.5 (Fig. S3b) was drastically different compared to that at room temperature, with conversion increasing from ~ 4% to 100% followed by an increase in selectivity towards the formation of *n*-butane (Table 4). We propose that this change in activity for Pd/Si_3_N_4_, particularly the increased selectivity towards *n*-butane, is a consequence of PdH phase formation which occurs more readily at the higher temperature (353 K vs. the room temperature test).

**Table 4:** Catalytic activity/selectivity comparison between samples with similar Pd particle size but using different support, i.e. SiO_2_, Si_3_N_4_ and Al_2_O_3_, at 1.5 h time on stream

Sample	Conversion (%)	*n*-Butane selectivity (%)	*Trans*-2-butene selectivity (%)	1-Butene selectivity (%)	*Cis*-2-butene selectivity (%)
Pd/Al_2_O_3_ 30-8.5	100	100	0	0	0
Pd/Si_3_N_4_ 30-8.5	100	48.2	35.7	2.4	13.6
Pd/SiO_2_ 30-8.5	20.1	0	31.2	64.3	4.5

#### Effect of Particle Size

Regarding the particle size effect, it appears that the profile previously observed (Pd/SiO_2_ 1.1 > 2.6 > 1.9 nm) was kept even when raising the temperature, albeit the difference in catalytic performance shown in Table 5 and Fig. S4 (i.e. from ~ 31 to 100% for Pd/SiO_2_ 16-3.5 and from ~ 12 to ~ 90% for Pd/SiO_2_ 175-70). However, the selectivity changed quite dramatically for Pd/SiO_2_ 175-70 and 16-3.5. The major product appeared to be the over-hydrogenated *n*-butane, possibly due the higher reaction rate caused by the higher temperature. Moreover, *trans*-2-butene became the predominant product among butenes, suppressing the production of *cis*-2-butene and 1-butene. Interestingly this behaviour was not seen for Pd/SiO_2_ 30-8.5. This could be related to the lower catalytic activity, which, other than preventing the formation of *n*-butane, causes the selective formation of 1-butene.

**Table 5:** Catalytic activity/selectivity comparison between samples supported on SiO_2_ but different particle size (1.1, 1.9, 2.7 nm respectively) after 1.5 h

Sample	Conversion (%)	*n*-Butane selectivity (%)	*trans*-2-butene selectivity (%)	1-butene selectivity (%)	*cis*-2-butene selectivity (%)
Pd/SiO_2_ 16-3.5	100	53.9	31.7	2.5	11.9
Pd/SiO_2_ 30-8.5	19.1	0	31.6	63.9	4.4
Pd/SiO_2_ 175-70	89.5	54.4	28.5	7.0	10.1

### In Situ XAFS

The 1,3-butadiene hydrogenation was followed using X-ray absorption spectroscopy in order to identify any evolution of the Pd nanoparticles under reaction conditions.

**Fig. 6:**
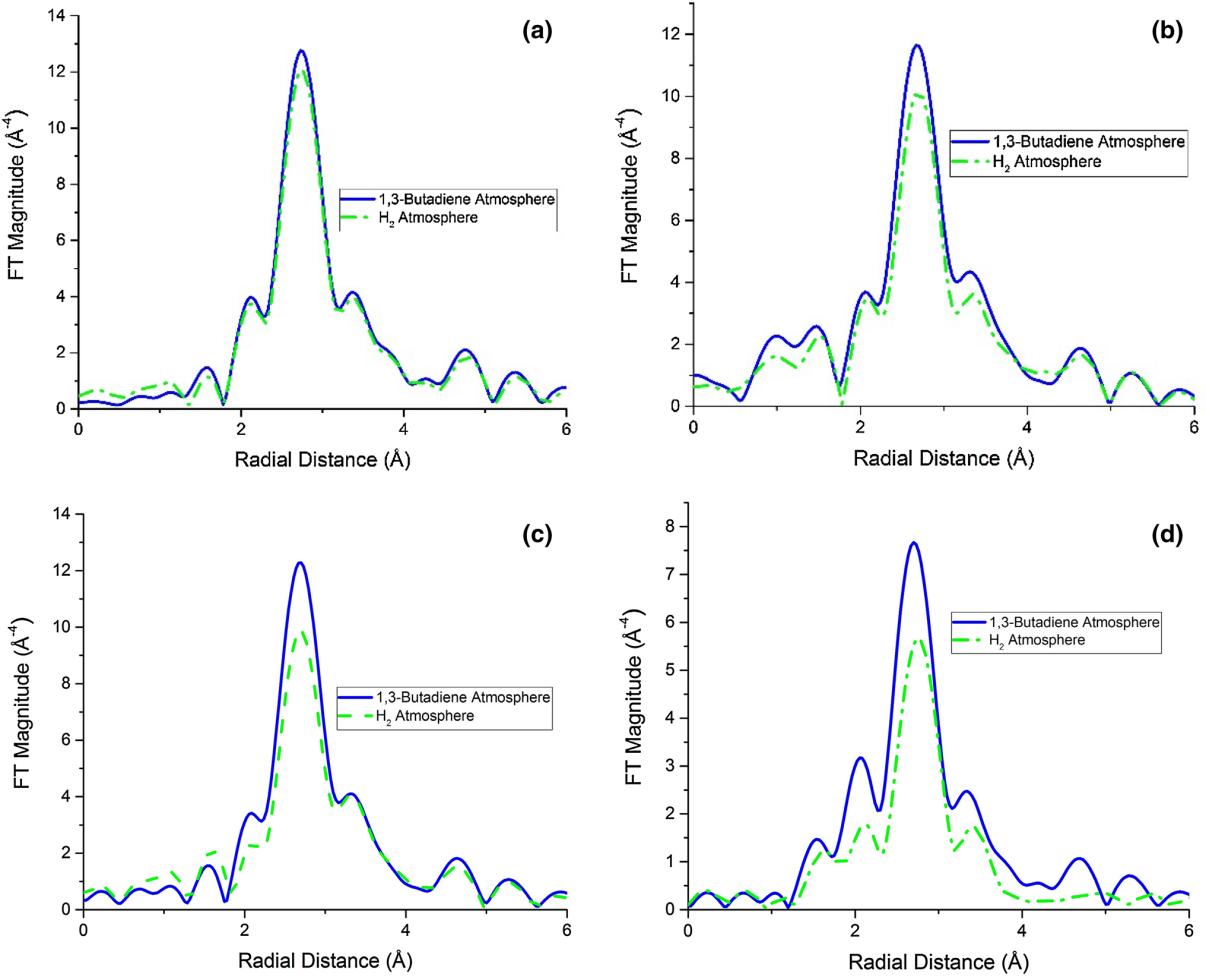
k^3^-weighted Fourier transform of: **a** Pd/SiO_2_ 30-8.5; **b** Pd/SiO_2_ 16-3.5; **c** Pd/Si_3_N_4_ 30-8.5; **d** Pd/Al_2_O_3_ 30-8.5; under two different atmospheres, hydrogen and 1,3-butadiene. As the gas composition changed an increase in the intensity of the Pd–Pd scattering first shell contribution was observed

A clear change in the EXAFS was seen with the changes in the FT as the gas composition varied, as shown in Fig. 5 for Pd/SiO_2_ 175-70. As the composition changed from H_2_ (pink solid line in the figure) to 1,3-butadiene (solid black line), a sizeable increase (14%) in the first shell FT intensity appeared. Subsequently after the introduction of the reaction mixture, a minor decrease by 3% of FT intensity occurred, followed by a further 3% decrease as the gas atmosphere changed back to H_2_. The same behaviour was observed for all the other samples examined, as shown in Fig. 6, where the most significant FT intensity changes are shown, and that this change occurred from switching of the H_2_ atmosphere to 1,3-butadiene.

**Fig. 7:**
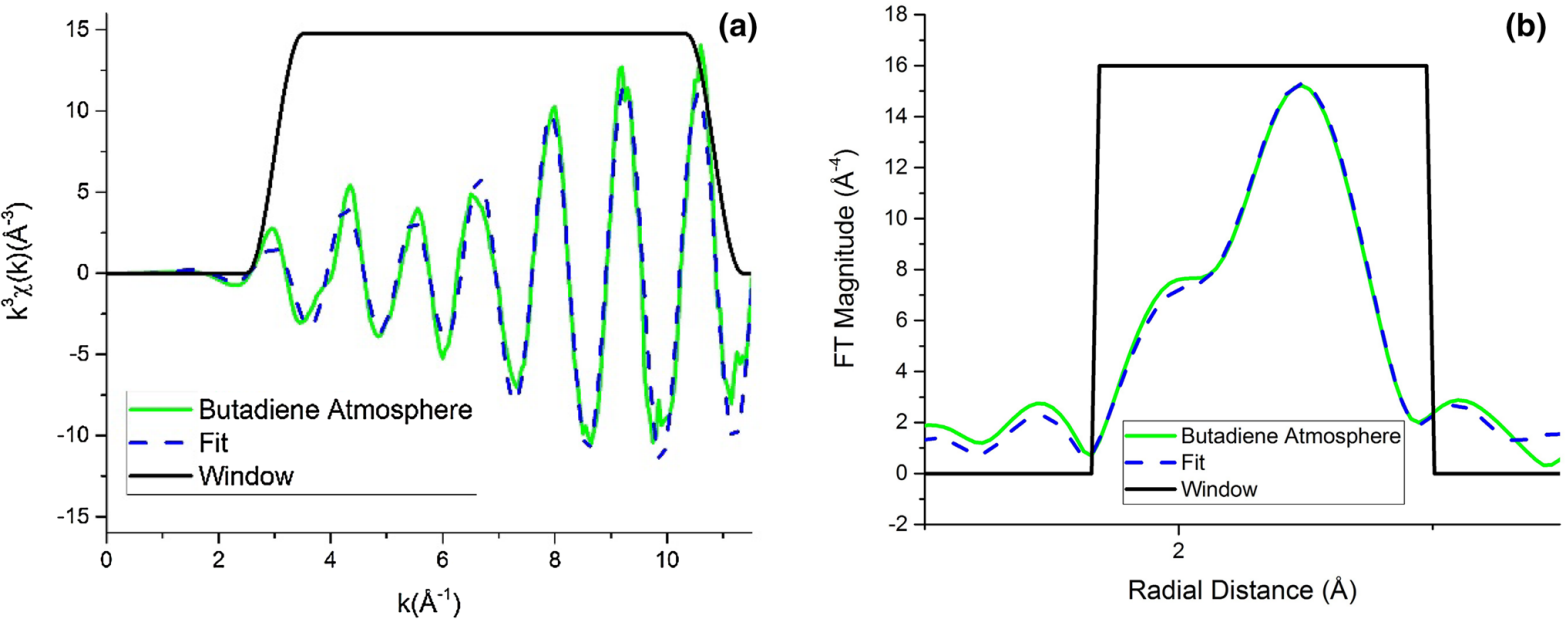
k^3^-weighted EXAFS fit in k (on the left) and R (on the right) space of Pd/SiO_2_ 175-70 under 1,3-butadiene atmosphere

In order to understand the changes, a fit of the data was needed. In Fig. 7 is shown one example of the results of an EXAFS fit for Pd/SiO_2_ 175-70, to show the good agreement between the experimental and simulated data. The other fit results are shown in Figs. S5, S6, S7, S8, S9, S10 in the ESI. In Tables S1, S2, S3, S4, S5 are summarized the results obtained from the fit and it can be seen that for all catalysts, in the presence of 1,3-butadiene, an increase in the 1st shell coordination (1CN) number and a slight decrease in the Pd–Pd bond distance occurred. This, we propose, could be explained by a removal of palladium hydride (PdH) from the Pd nanoparticles. The reason for this increase can be attributed to the removal of the interference between Pd and PdH EXAFS signals, as illustrated in Fig. S10. The EXAFS signal for the PdH phase has a lower amplitude and is heavily out of phase with Pd^0^ scattering phase, leading to a reduction in the total EXAFS amplitude. Hence the presence of PdH is strongly inferred by the changing FT intensities with/without hydrogen flowing. As can be seen from Table 6, under reaction conditions (H_2_/1,3-butadiene) the 1st coordination number (1CN) clearly decreases. A reduction in FT intensity for the Pd–Pd contribution is seen under reaction conditions suggestive of the (re)appearance of the hydride phase, the extent of its formation seemingly mitigated by the presence of 1,3-butadiene during the reaction process.

**Table 6:** First shell (1CN) for Pd supported catalysts, kept under steady state conditions for 20 min, under H_2_ and/or 1,3-butadiene atmosphere(s)

Gas composition\sample name	Pd/SiO_2_ 30-8.5	Pd/SiO_2_ 175-70	Pd/SiO_2_ 16-3.5	Pd/Si_3_N_4_ 30-8.5	Pd/Al_2_O_3_ 30-8.5
Hydrogen	9.76 ± 0.42	9.59 ± 0.53	8.56 ± 0.58	8.86 ± 0.42	5.35 ± 0.69
1,3-Butadiene	10.32 ± 0.44	10.94 ± 0.81	10.14 ± 0.88	10.89 ± 0.61	7.27 ± 2.79
Hydrogen/1,3-butadiene	10.16 ± 0.36	10.63 ± 0.31	10.06 ± 0.48	10.84 ± 0.45	7.65 ± 0.69
Hydrogen (after reaction)	10.06 ± 0.45	10.33 ± 0.43	9.73 ± 0.35		6.72 ± 0.53

When hydrogen flows again over each catalyst a subsequent increase of the bond distance and decrease of 1CN is again observed suggestive of reformation of the hydride phase. Whilst all catalysts behaved the same way, the changes observed in the Al_2_O_3_-supported catalyst were more dramatic. First of all, the initial coordination number obtained was much smaller than that obtained from the other samples; whereas this could suggest the presence of a large number of very small nanoparticles (or even atomically dispersed Pd), the most likely reason is a large amount of palladium hydride present.

**Fig. 8:**
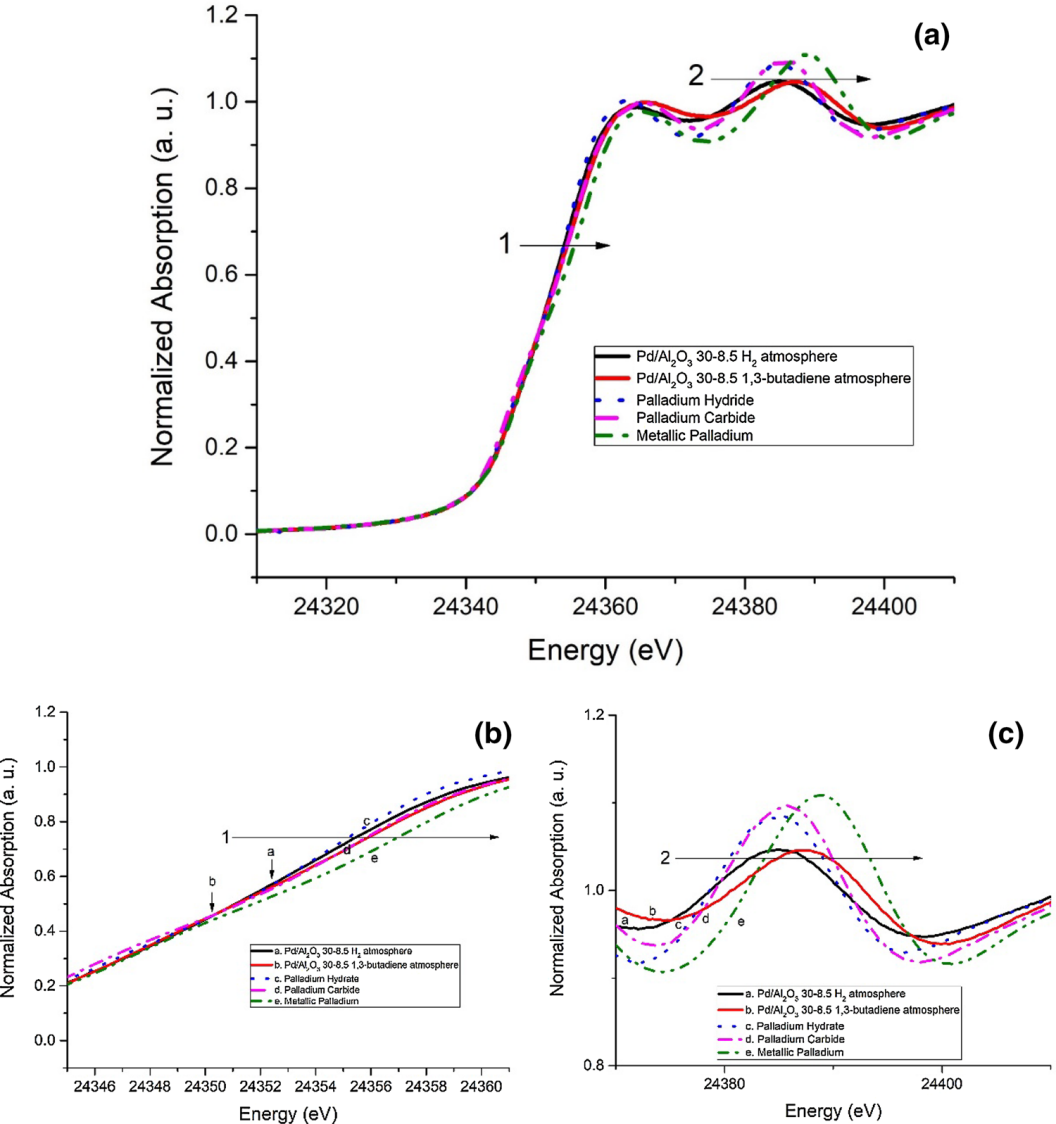
**a** XANES spectra of Pd/Al_2_O_3_ 30-8.5 under H_2_ and 1,3-butadiene, compared to Pd^0^ and PdH reference. Arrows 1 and 2 indicate the shift towards higher energy of the Pd K-edge signal due to hydride and carbide formation; **b** close-up of the edge shift indicated by arrow 1; **c** close-up of the feature indicated by arrow 2

The accompanying XANES spectra of Pd/Al_2_O_3_ 30-8.5, shown in Fig. 8, appeared to confirm the presence of PdH in the sample when it was under H_2_, due to the characteristic edge shift towards higher energy compared to metallic palladium indicated in the figure by arrow 1, as shown by Bugaev et al. [24]. This was further evidenced by the shift of the first multiple scattering component of the XANES (indicated by arrow 2 in the figure) due to the different scattering contribution of PdH. However, when the Al_2_O_3_ supported sample was kept under 1,3-butadiene atmosphere a further shift towards higher energy appeared which suggested the formation of palladium carbide. This was further indicated by a strong shift of the multiple scattering component (arrow 2) towards higher energy which suggested a noticeable formation of PdC and the subsequent change in the scattering contribution. The high amount of PdC caused a reduction in the intensity of the EXAFS signal (particularly Pd–Pd contribution in the FT) for the same reason as formation of PdH does [24].

## Discussion

The catalytic results obtained here match well with previous reported literature; a study by Garcia Cervantes et al., similar to the one here reported, showed the same support influence using the supports examined here [25]. This was inclusive of a similar deactivation effect with TOS although the time required for deactivation for the samples used in this work was much shorter, roughly a third of what was reported using similar reaction conditions albeit the Garcia Cervantes study used a slightly lower H_2_/1,3-butadiene ratio (87/13) compared to the ones used here (98/2). The authors speculated that the deactivation was due to the formation of carbonaceous species on the surface of the nanoparticles. The role of these carbonaceous species on the selectivity of the Pd nanoparticles is still under discussion; according to some studies, a pre-treatment of 1,3-butadiene leads to a sharp decrease in activity and to a higher quantity of *n*-butene produced due to formation of PdC (thought to be at the surface), identified through the expansion of the Pd lattice by XRD [26, 27]. However, another study showed that in a fixed bed reactor at 298 K and using a molar ratio of the reaction mixture 1:1.2 1,3-butadiene/H_2_, a greater amount of 1-butene was observed with time, which was thought to be due to butadiene oligomers forming a carbonaceous overlayer and increasing this selectivity, albeit with a reduction of activity [28]. The reason for this variation in results using similar catalysts might lie in the different reaction conditions applied in both cases: batch [26, 27] and fixed bed reactor [28] studies. The presence of palladium carbide was shown to apply a dramatic effect in increasing the amount of sub-surface palladium hydride, which has shown to be a key factor in the hydrogenation of butenes [21]. It reasonable to assume that in a batch reaction the longer retention time of hydrocarbon on the surface causes the formation of a carbonaceous layer, thus causing an over-hydrogenation of the butenes.

**Fig. 9:**
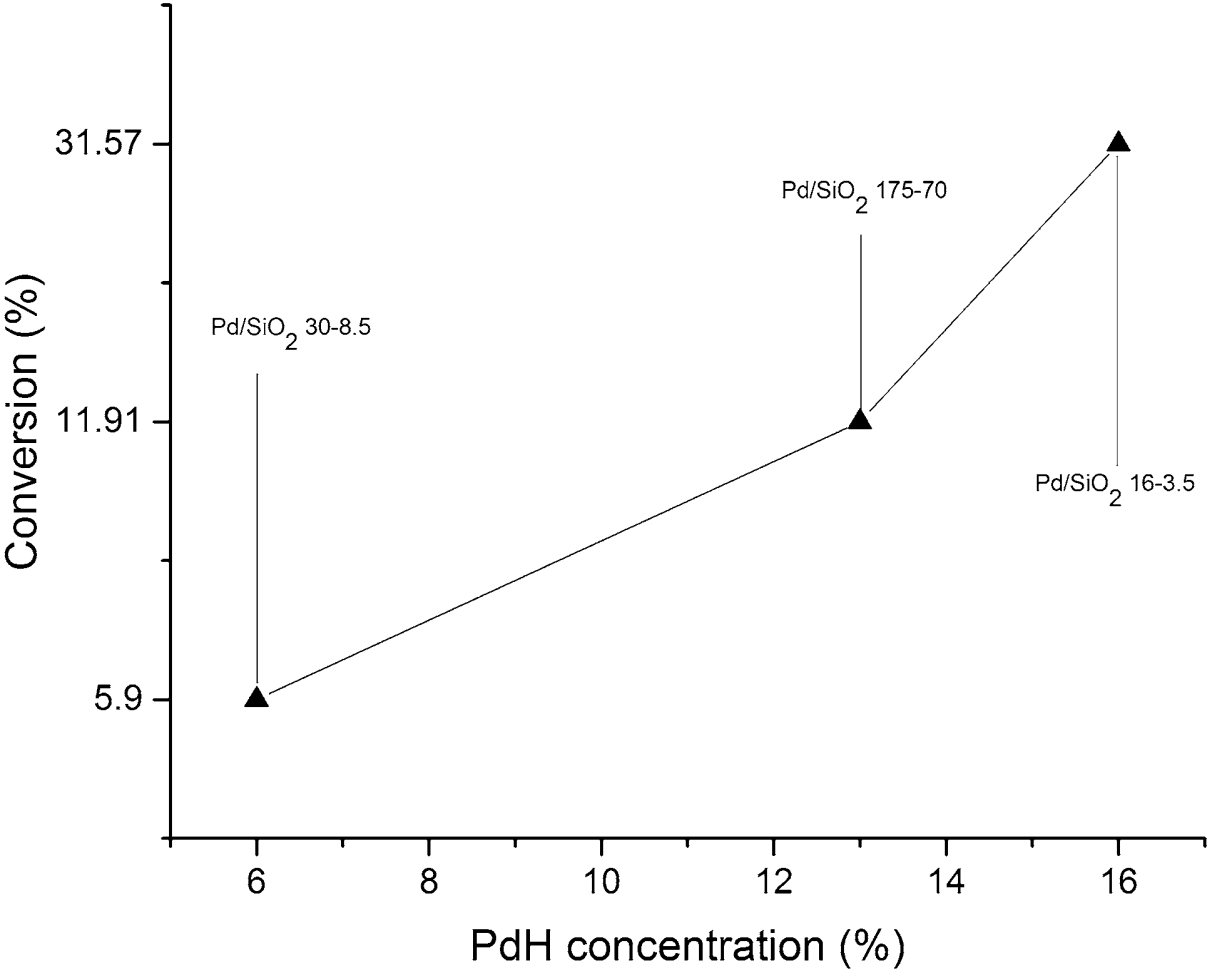
Variation of conversion as function of the PdH concentration for Pd/SiO_2_ supported samples at 298 K

The importance of PdH in the formation of *n*-butene can be seen through the EXAFS results obtained. The amount of PdH present in the samples, in particular in the case of Pd/SiO_2_, can be directly correlated to the catalytic activity as shown by Fig. 9 and Fig. S11. A greater presence of hydride corresponds to a greater butadiene conversion to butane. In a previously reported study on the isomerisation and hydrogenation of *cis*-2-butene, it was shown that while the hydrogenation activity decreased, the isomerisation rate remained constant [21, 29]. The authors attributed this behaviour to a lack of hydrogen capable of reacting with the carbon–carbon double bond. As time on stream increased the hydrogenation became selectively suppressed because bulk H species are consumed and are not replenished under steady-state conditions. Is therefore plausible that in this previous study that the presence of interstitial hydrogen was directly correlated to the catalytic conversion and its removal preventing the formation of *n*-butane. This phenomenon was clearly visible in the Pd/Al_2_O_3_ 30-8.5 sample, where the large amount of PdH, even after being under a 1,3-butadiene atmosphere for ~ 30 min. It appears that Al_2_O_3_ is able to promote significant formation of PdH which leads to an extensive formation of *n*-butane. However, amongst the other samples, the behaviour of Pd/Si_3_N_4_ 30-8.5 appeared to be an outlier; whereas the amount of hydride found was higher than for the SiO_2_ samples, the activity was actually the lowest. This could be explained by suggesting that the support plays more of a role in the catalytic process than just to facilitate PdH formation. The results obtained from the Pd/SiO_2_ 30-8.5 shows catalytic performances more akin to the Si_3_N_4_ support sample rather than the Al_2_O_3_ one, albeit with a slightly higher catalytic activity. The different behaviour observed for SiO_2_ and Si_3_N_4_ supported samples, compared to Al_2_O_3_, could be caused by the lack of replenishment of interstitial hydrogen and paired with a lack in the formation of PdC, possibly due a different metal-support interaction. In general, it appears that the presence of PdH (possibly in conjunction with PdC) is detrimental to the selectivity towards butenes, causing a higher production of *n*-butane; working on the basis that excessive PdC formation is detrimental to catalyst performance in general, it can be assumed that the active phase toward the production of butenes is metallic Pd as shown by the higher selectivity of SiO_2_ and Si_3_N_4_ supported samples. This is further highlighted in the catalytic results obtained at 353 K, where Pd/Al_2_O_3_ appears to produce only *n*-butane due to the high amount of PdH still present in the system. Interestingly, the activity/selectivity for Si_3_N_4_ 30-8.5 changes quite dramatically between room temperature and 353 K, with a drastic improvement in the activity (from 4 to 100%) but a drop in selectivity (the amount of *n*-butane formed went from 0 to ~ 48%). However, it has to be noted that these results correlate well with the EXAFS results here obtained, which see Pd/Si_3_N_4_ presenting a larger increase, compared to the SiO_2_ supported samples, to its coordination number when the atmosphere is switched from H_2_ to 1,3-butadiene. A possible explanation for the different behaviour of Pd/Si_3_N_4_ 30-8.5 between 298 and 353 K could be attributed to a higher activation barrier required for bulk PdH formation on the nanoparticles. This explain the low deactivation time at low temperature as only surface PdH is formed and is quickly depleted.

It appears also that particle size affects the behaviour of Pd supported catalysts for 1,3-butadiene hydrogenation. Small nanoparticles (Pd/SiO_2_ 16-3.5 ~ 1 nm), which possess a higher amount of undercoordinated atoms, appear the most active overall. We note that Silvestre-Alberto et al. observed an increasing TOF (linear response) with increased particle size, which they attributed to the presence of increasingly larger low-index facets [6, 7]. One could therefore envisage that the sample Pd/SiO_2_ 30-8.5, possessing an intermediate particle size, would have a reduced number of undercoordinated atoms than Pd/SiO_2_ 16-3.5 and yet smaller [110] facets than Pd/SiO_2_ 175-70 and hence the catalytic performance is somewhere in between. However it has not been possible to rule out that the Pd/SiO_2_ 30-8.5 may be compromised in performance by the presence of unexpected contamination [6].

## Conclusions

Due to the synthesis method, which allowed a high control over the particle size of the nanoparticles, a clear understanding of the effect of particle size and support effects for Pd nanoparticle in the hydrogenation of 1,3-butadiene has been reached, and in particular we observe:

1. a clear correlation between the presence of the hydride phase and the catalytic behaviour of the system; the presence of a large amount of PdH leads to catalysts exhibiting high conversion and the formation of over-hydrogenated species (*n*-butane), particularly in the case of the Al_2_O_3_ and Si_3_N_4_ supported samples;
2. the active phase toward the selective production of butenes appears to be metallic palladium, being the only phase detected in the more selective SiO_2_ and Si_3_N_4_ supported samples. However, the higher selectivity observed is coincident with a lower overall activity of these samples;
3. the support plays an important role in the activity of the catalyst as it can be seen by the low activity, but higher selectivity, of the Si_3_N_4_ supported sample (~ 4% conversion at a later stage of deactivation) at room temperature, and the SiO_2_ sample becoming more selective due the lower formation of PdH.
4. particle size plays also an important role in determining the catalytic activity of Pd NPs. The observation of a reverse volcano plot (for size vs. activity) can be rationalised in terms of a combination of a very high surface area of very small nanoparticles (1.1 nm) or else to the presence of highly-active, low-index facets, [i.e. (110) and (111)], for nanoparticles 2.6 nm in size. The catalyst (Pd/SiO_2_ 30-8.5) which possesses an intermediate particle size (i.e. with a lower surface area than the smallest particle but with smaller low-index facets than the largest particle size) shows the least activity.

## Electronic supplementary material

Below is the link to the electronic supplementary material.

Supplementary material 1 (DOCX 1439 KB)
