# Supplementary material for: Effect of Particle Size and Support Type on Pd Catalysts for 1,3-Butadiene Hydrogenation
Source: Top Catal. 2018 Jan 19;61(3):162–74. doi: 10.1007/s11244-018-0887-4 (PMC6413808; doi:10.1007/s11244-018-0887-4)
Supplement: Supplementary file 1 — Supplementary material 1 (DOCX 1439 KB) [file 11244_2018_887_MOESM1_ESM.docx]

**Electronic Supporting Information for**

**Effect of particle size and support type on Pd catalysts for 1,3-butadiene hydrogenation**

Donato Decarolis^a,b^, Ines Lezcano-Gonzalez^a,b^, Diego Gianolio^c^, Andrew M. Beale^a,b,*^

by

a University College London, Chemistry Department, 20 Gordon Street, London, WC1H 0AJ, UK

b Research Complex at Harwell, Rutherford Appleton Laboratory, Harwell Science and Innovation Campus, Harwell Didcot, Oxon, OX11 0FA, UK

c Diamond Light Source Ltd, Harwell Science and Innovation Campus, Chilton, Didcot OX11 0DE, UK.

**TEM results**

| 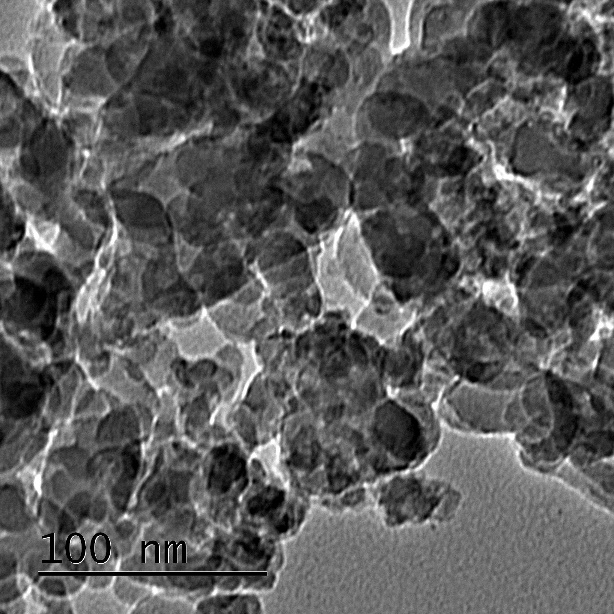 | 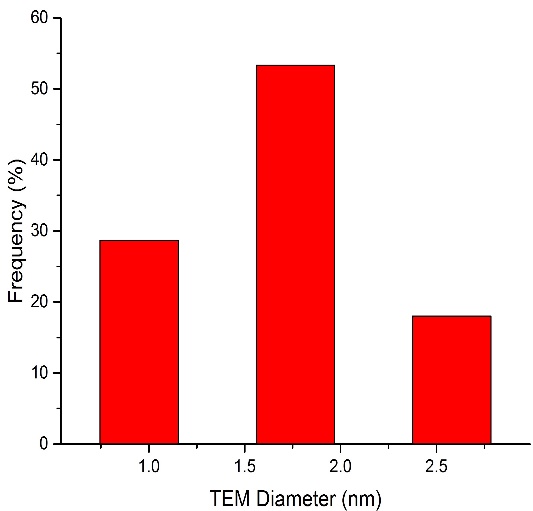 | |
| --- | --- | --- |
|  | Mean particle size (nm) | 1.9 |
|  | Standard Deviation | 0.3 |
| **Figure S1.** On the left: TEM micrograph of Pd/SiO_2_ 30-8.5 samples; on the right: particle size distribution (150 particles counted towards the particle size distribution) | | |

| 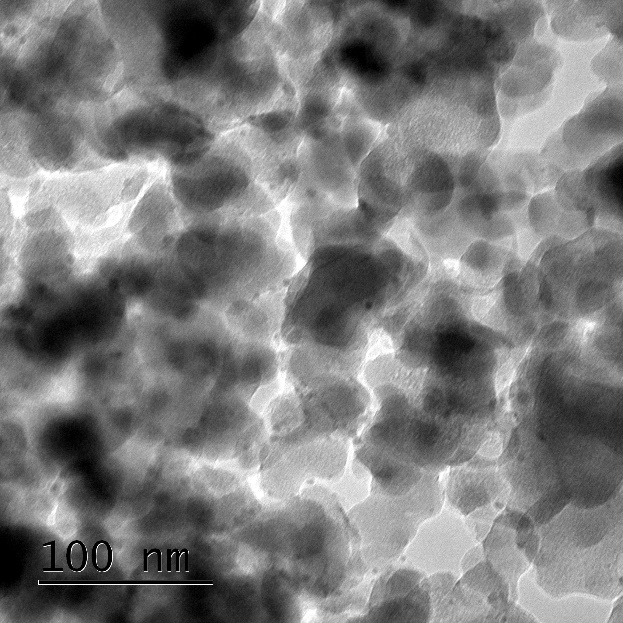 | 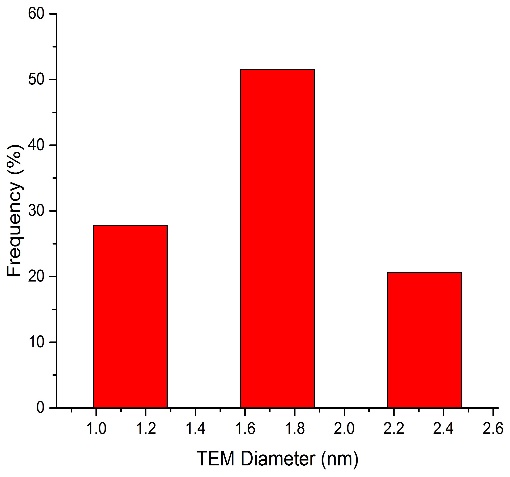 | |
| --- | --- | --- |
|  | Mean particle size (nm) | 2.07 |
|  | Standard Deviation | 0.51 |
| **Figure S2.** On the left: TEM micrograph of Pd/Si_3_N_4_ 30-8.5 samples; on the right: particle size distribution (150 particle counted toward particle size distribution) | | |

**Catalytic Results**

| 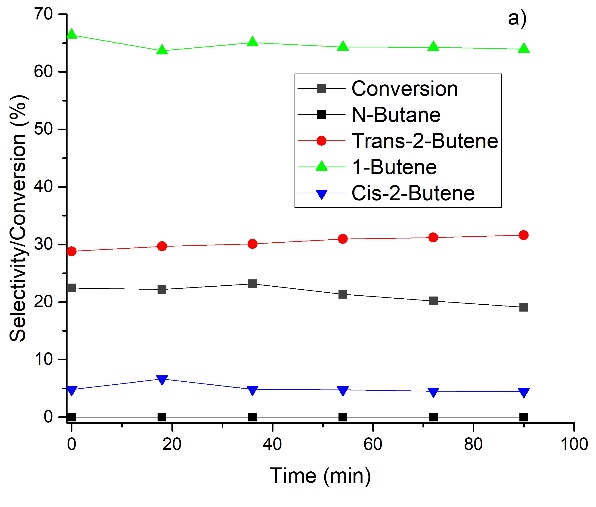 | 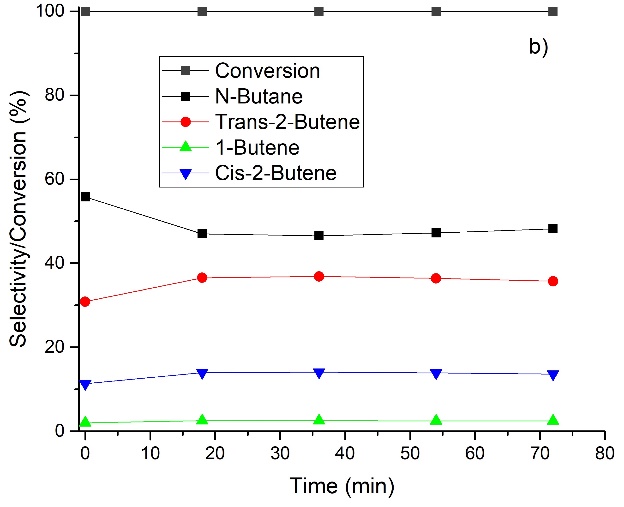 |
| --- | --- |
| **Figure S3.** Evolution of conversion and selectivity for 1,3-butadiene hydrogenation over a) Pd/SiO_2_ 30-8.5; b) Pd/Si_3_N_4_ 30-8.5 as a function of time on stream | |

| 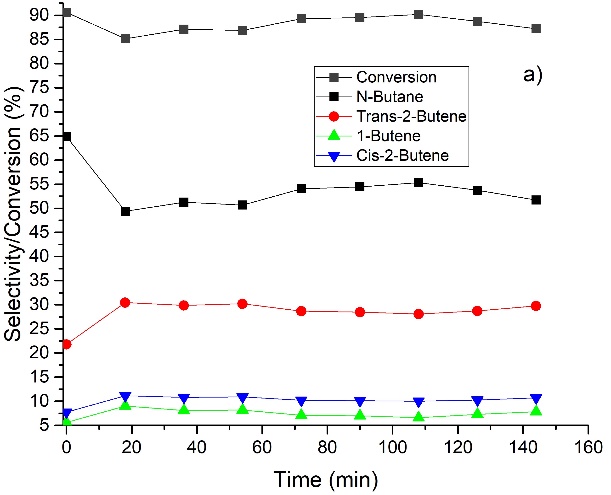 | 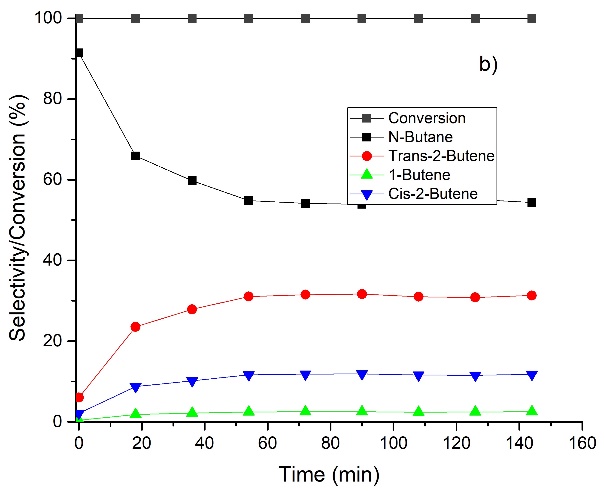 |
| --- | --- |
| **Figure S4.** Evolution of conversion and selectivity for 1,3-butadiene hydrogenation over a) Pd/SiO_2_ 175-70; b) Pd/SiO_2_ 16-3.5 as a function of time on stream | |

**EXAFS results**

| 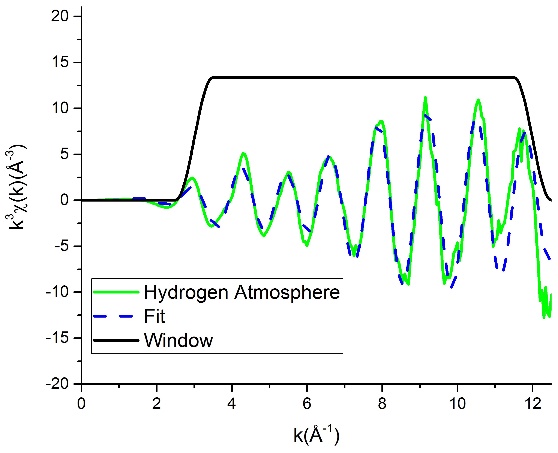 | 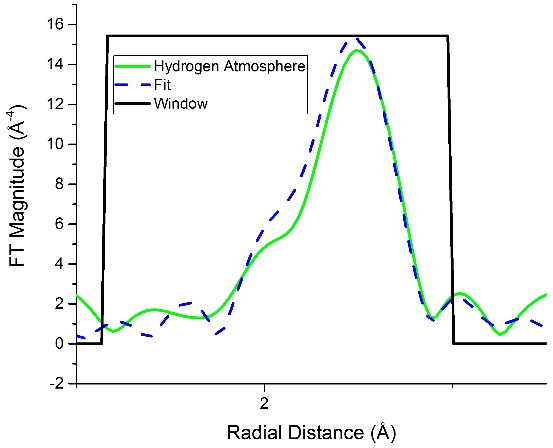 |
| --- | --- |
| **Figure S5.** k^3^-weighted EXAFS fit in k (on the left) and R (on the right) space of Pd/SiO_2_ 175-70 under H_2_ atmosphere | |

| 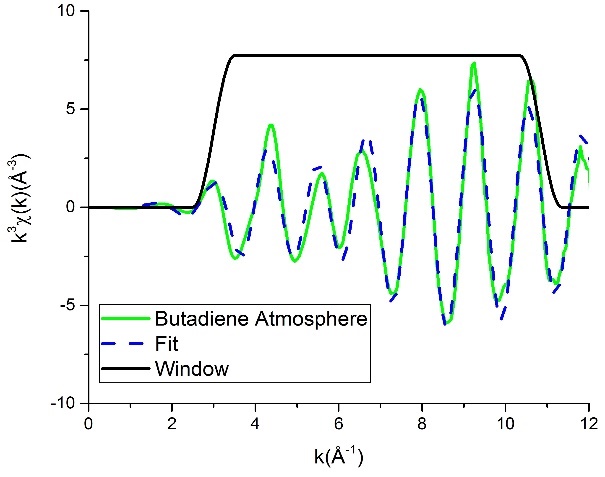 | 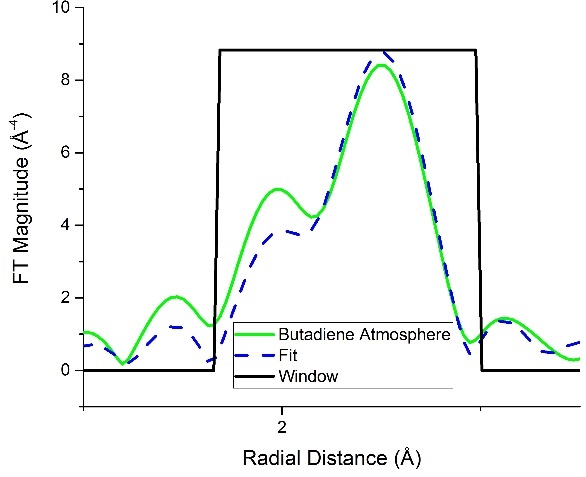 |
| --- | --- |
| **Figure S6.** k^3^-weighted EXAFS fit in k (on the left) and R (on the right) space of Pd/Al_2_O_3_ 30-8.5 under 1,3-butadiene atmosphere | |

| 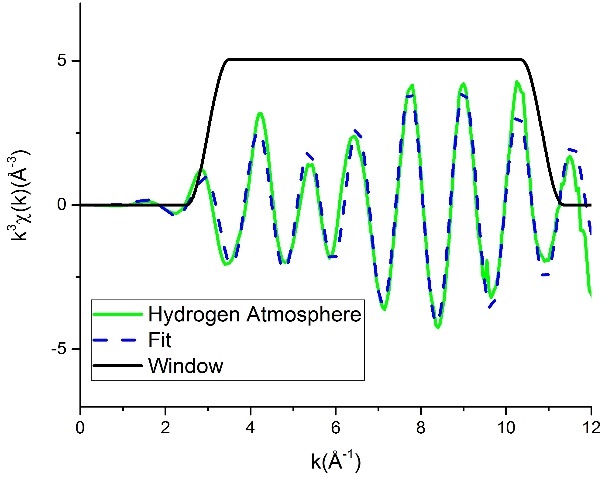 | 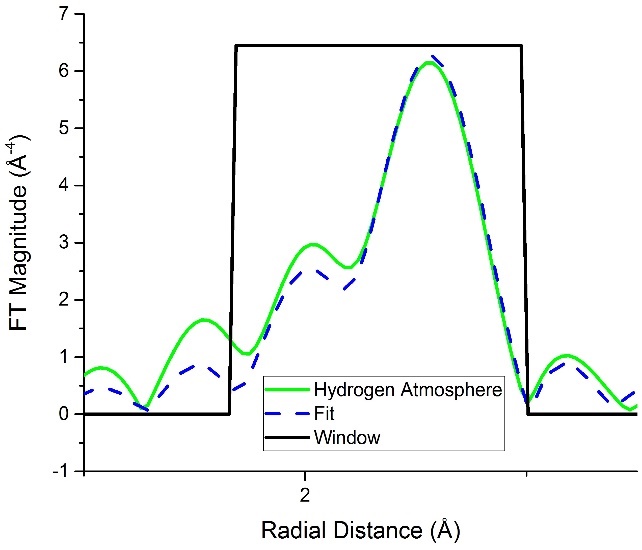 |
| --- | --- |
| **Figure S7.** k^3^-weighted EXAFS fit in k (on the left) and R (on the right) space of Pd/Al_2_O_3_ 30-8.5 under H_2_ atmosphere | |

| 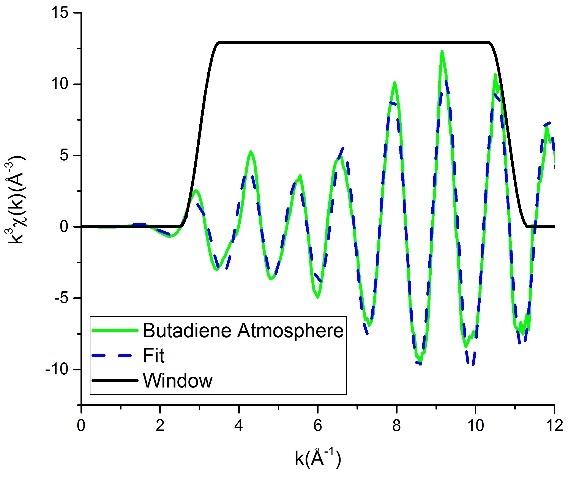 | | 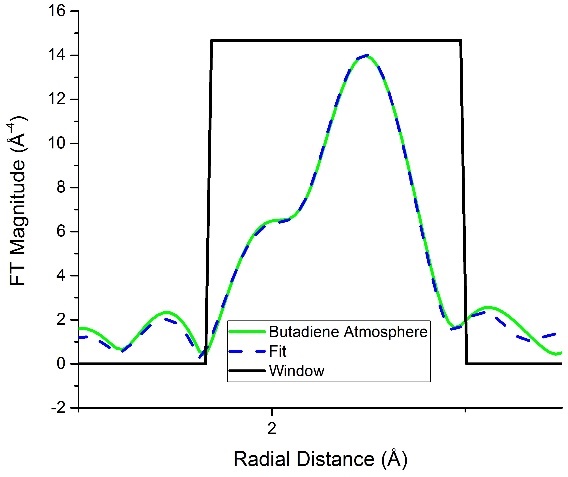 |
| --- | --- | --- |
| **Figure S8.** k^3^-weighted EXAFS fit in k (on the left) and R (on the right) space of Pd/Si_3_N_4_ 30-8.5 under 1,3-butadiene atmosphere | | |
| 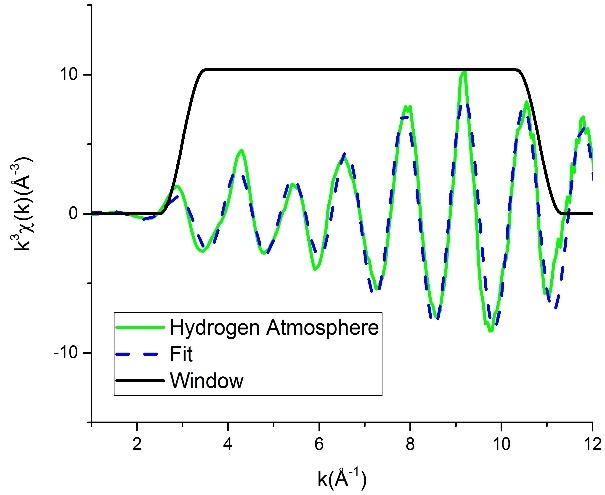 | 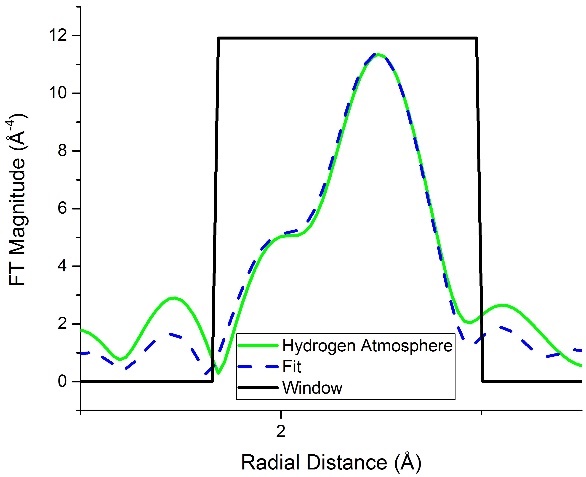 | |
| **Figure S9.** k^3^-weighted EXAFS fit in k (on the left) and R (on the right) space of Pd/Si_3_N_4_ 30-8.5 under H_2_ atmosphere | | |

| 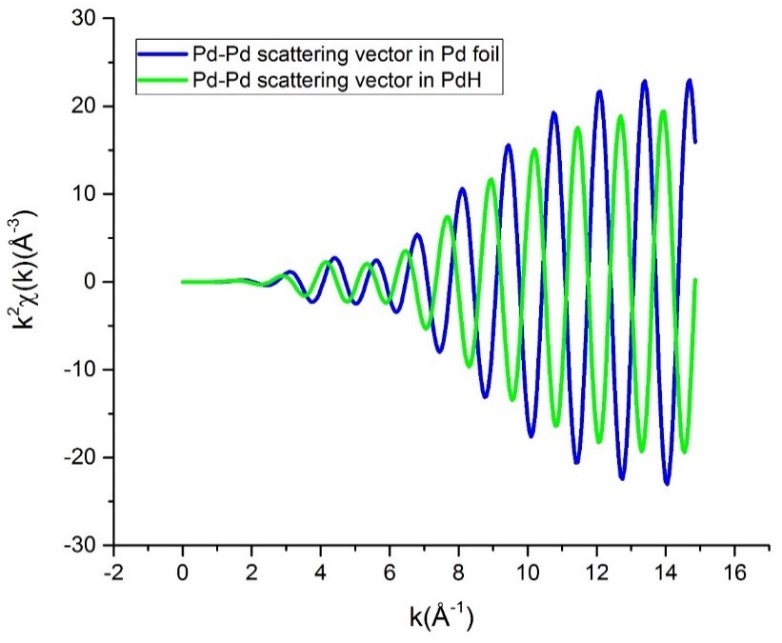 |
| --- |
| **Figure S10.** k^2^-EXAFS for Pd-Pd scattering vector in a Pd foil and in PdH calculated from structural parameter through IEFFIT. The two scattering vector are out of phase, leading to phase cancellation. |

| **Table S1** **EXAFS fit results obtained from Pd/SiO_2_ 30-8.5, using a constant Debye-Waller factor, as a function of the gas composition** | | | | |
| --- | --- | --- | --- | --- |
| **Gas composition** | **1CN** | **1^st^  shell radius ( Å)** | **ΔE** | **R_factor_** |
| Hydrogen | 9.76 ± 0.42 | 2.745 ± 0.004 | -4.0 ± 0.6 | 0.021 |
| 1,3-Butadiene | 10.32 ± 0.44 | 2.737 ± 0.002 | -3.8 ± 0.3 | 0.004 |
| Hydrogen/ 1,3-Butadiene | 10.16 ± 0.36 | 2.742 ± 0.004 | -3.8 ± 0.5 | 0.013 |
| Hydrogen (After reaction) | 10.06 ± 0.45 | 2.744 ± 0.005 | -5 ± 0.5 | 0.023 |

| **Table S2. EXAFS fit results obtained from Pd/SiO_2_ 175-70, using a constant Debye-Waller factor, as a function of the gas composition** | | | | |
| --- | --- | --- | --- | --- |
| **Gas composition** | **1CN** | **1^st^  shell radius ( Å)** | **ΔE** | **R_factor_** |
| Hydrogen | 9.59 ± 0.53 | 2.746 ± 0.006 | -5.11 ± 0.85 | 0.009 |
| 1,3-Butadiene | 10.94 ± 0.81 | 2.740 ± 0.004 | -5.04 ± 0.52 | 0.002 |
| Hydrogen/ 1,3-Butadiene | 10.63 ± 0.31 | 2.744 ± 0.003 | -5.12 ± 0.44 | 0.002 |
| Hydrogen (After reaction) | 10.33 ± 0.43 | 2.743 ± 0.0045 | -4.87 ± 0.63 | 0.005 |

| **Table S3. EXAFS fit results obtained from Pd/SiO_2_ 16-3.5, using a constant Debye-Waller factor, as a function of the gas composition** | | | | |
| --- | --- | --- | --- | --- |
| **Gas composition** | **1CN** | **1^st^  shell radius ( Å)** | **ΔE** | **R_factor_** |
| Hydrogen | 8.56 ± 0.58 | 2.746 ± 0.007 | -4.64 ± 0.97 | 0.015 |
| 1,3-Butadiene | 10.14 ± 0.88 | 2.738 ± 0.004 | -4.28 ± 0.62 | 0.003 |
| Hydrogen/ 1,3-Butadiene | 10.06 ± 0.48 | 2.736 ± 0.005 | -4.54 ± 0.7 | 0.007 |
| Hydrogen (After reaction) | 9.73 ± 0.35 | 2.743 ± 0.004 | -4.51 ± 0.51 | 0.004 |

| **Table S4. EXAFS fit results obtained from Pd/Si_3_N_4_ 30-8.5, using a constant Debye-Waller factor, as a function of the gas composition** | | | | |
| --- | --- | --- | --- | --- |
| **Gas composition** | **1CN** | **1^st^  shell radius ( Å)** | **ΔE** | **R_factor_** |
| Hydrogen | 8.86 ± 0.42 | 2.747 ± 0.005 | -6.41 ± 0.68 | 0.007 |
| 1,3-Butadiene | 10.89 ± 0.61 | 2.739 ± 0.003 | -5.895 ± 0.402 | 0.001 |
| Hydrogen/ 1,3-Butadiene | 10.84 ± 0.45 | 2.745 ± 0.004 | -5.8 ± 0.61 | 0.006 |

| **Table S5. EXAFS fit results obtained from Pd/Al_2_O_3_ 30-8.5, using a constant Debye-Waller factor, as a function of the gas composition** | | | | |
| --- | --- | --- | --- | --- |
| **Gas composition** | **1CN** | **1^st^  shell radius ( Å)** | **ΔE** | **R_factor_** |
| Hydrogen | 5.35 ± 0.69 | 2.752 ± 0.007 | -7.49 ± 1.11 | 0.018 |
| 1,3-Butadiene | 7.27 ± 2.79 | 2.737 ± 0.004 | -7.75 ± 0.62 | 0.004 |
| Hydrogen/ 1,3-Butadiene | 7.65 ± 0.69 | 2.752 ± 0.007 | -6.07 ± 1.2 | 0.016 |
| Hydrogen (After reaction) | 6.72 ± 0.53 | 2.77 ± 0.01 | -8.09 ± 1.8 | 0.038 |

**Discussion**

| **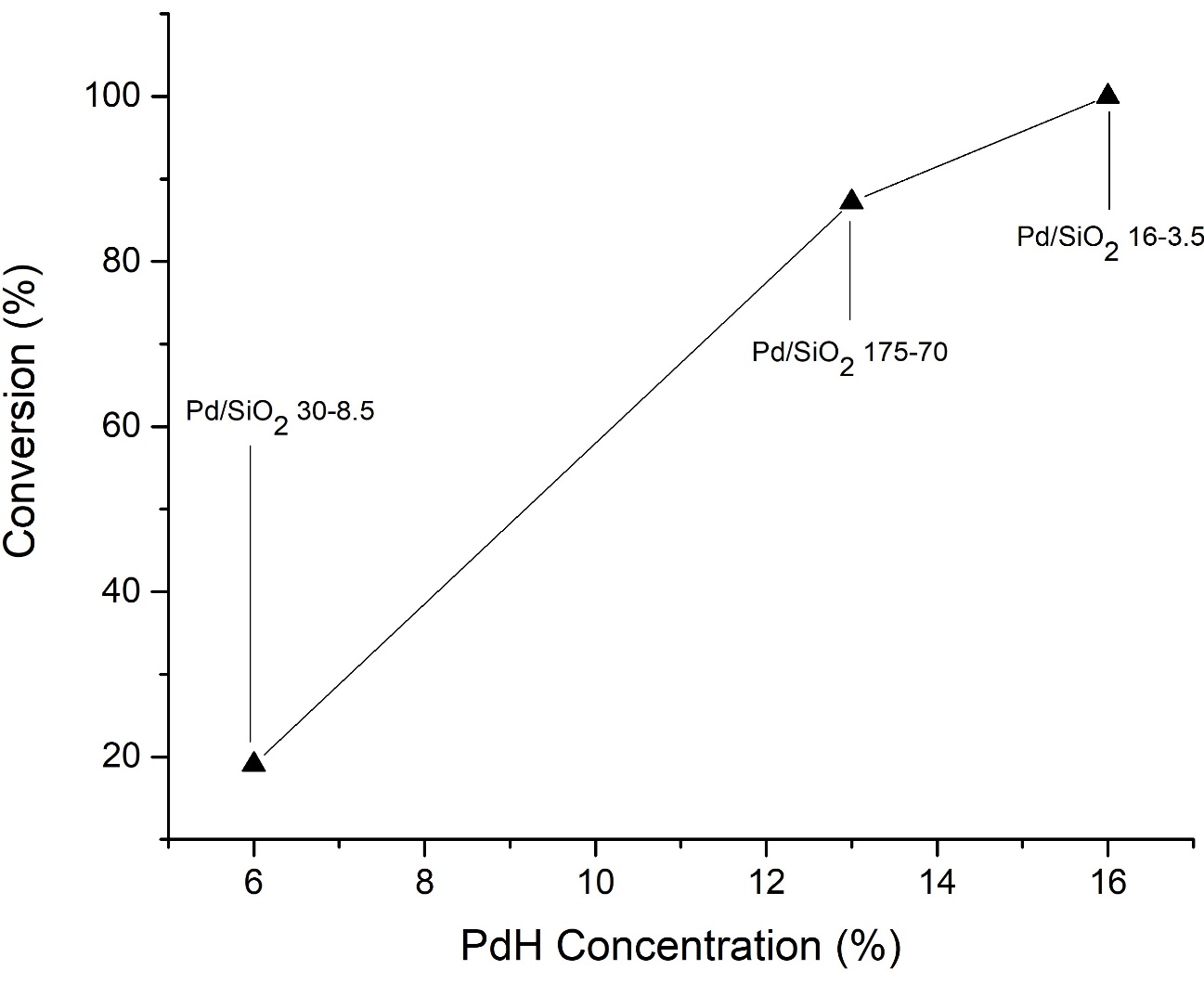** |
| --- |
| **Figure S11.** Variation of conversion as function of the PdH concentration for Pd/SiO_2_ supported samples at 353 K. |

Note: Copies of raw data can be found at: https://tinyurl.com/ButadieneHydrogPd2017
